# A Novel Heterozygous Pathogenic Variation in *CYCS* Gene Cause Autosomal Dominant Non-Syndromic Thrombocytopenia 4 in a Large Chinese Family

**DOI:** 10.3389/fgene.2021.783455

**Published:** 2022-01-18

**Authors:** Fengyu Che, Jiangang Zhao, Yujuan Zhao, Zhi Wang, Liyu Zhang, Ying Yang

**Affiliations:** ^1^ Shaanxi Institute for Pediatric Diseases, Xi’an Children’s Hospital, Xi’an, China; ^2^ Department of Neonatology, Xi’an Children’s Hospital, Xi’an, China

**Keywords:** CYCS gene, platelet counts, cytochrome c, mutation, thrombocytopenia

## Abstract

**Aim:** To determine the etiology of a Chinese family with thrombocytopenia by analyzing the clinical features and genetic variation.

**Methods:** Clinical profiles and genomic DNA extracts of the family members were collected for the study. Whole exome sequencing and Sanger sequencing was used to detect the associated genetic variation and verify the family co-segregation respectively. Bioinformatics analysis assessed the pathogenicity of missense mutations.

**Results:** The study reported a 3-generation pedigree including eight family members with thrombocytopenia. The platelet counts of the patients were varied, ranging from 38 to 110 × 10^9^/L (reference range: 150–450 x 10^9^/L). The mean volumes and morphology of the sampled platelet were both normal. The bleeding abnormality and mitochondriopathy were not observed in all the patients. Clinical signs of thrombocytopenia were mild. A novel heterozygous missense variant c.79C > T (p.His27Tyr) was identified in *CYCS* gene associated with autosomal dominant thrombocytopenia.

**Conclusion:** We report the first large family with autosomal dominant non-syndromic thrombocytopenia 4 in a Chinese family, a novel heterozygous missense variant c.79C > T (p.His27Tyr) was identified. The whole exome sequencing is an efficient tool for screening the variants specifically associated with the disease. The finding enriches the mutation spectrum of *CYCS* gene and laid a foundation for future studies on the correlation between genotype and phenotype.

## 1 Introduction

Thrombocytopenia is defined as a haematological condition with blood platelet count below 100–150 × 10^9^/L (Greenberg and Kaled, 2013). It is correlated with inherited hematologic diseases, immune diseases (e.g.rheumatoid), radiation/chemotherapy injuries, infection, and drug-related thrombocytopenia (Lee and Lee, 2016; Franchini et al., 2017). At present, more than 20 genes have been reported to be associated with the incidence of inherited thrombocytopenia, including syndromic, and non-syndromic (Nurden and Nurden, 2020). Hence, understanding the disease from a genetic perspective is a key of providing effective diagnosis and prognostic risk assessment.

The *CYCS* gene encodes the Cytochrome C (Cyt-c), which is a small and stable heme protein with heme C as its auxiliary group (Stevens, 2011). The Cyt-c protein is an essential component anchoring in the inner membrane of the mitochondrion for multiple bio-functions (Santucci et al., 2019). It is mainly responsible of transferring electrons from cytochrome b to the cytochrome oxidase complex (Kagan et al., 2005; Li et al., 2006; Ow et al., 2008), and also could initiate cell apoptosis as an antioxidant agent (Wang et al., 2003; Zhao et al., 2003; Lee and Xu, 2007). It is reported that the heterozygous mutation of *CYCS* gene on chromosome 7p15 can caused autosomal dominant non-syndromic thrombocytopenia-4 (THC4, OMIM: 612004) (Morison et al., 2008). The clinical features of THC4 were characterized with mild thrombocytopenia, normal platelet size and morphology, and no increased bleeding tendency. So far, there are only 4 pedigrees with nonsyndrome thrombocytopenia and 1 pedigree with hemophilia A, associated with variants in *CYCS* gene (Morison et al., 2008; De Rocco et al., 2014; Johnson et al., 2016; Uchiyama et al., 2018; Turro et al., 2020). None of the patients in these reports showed symptoms resulting from cell apoptosis and abnormal mitochondrial oxidative respiratory chain, whereas thrombocytopenia was present.

We studied a first pedigree with autosomal dominant non-syndrome thrombocytopenia 4 in a Chinese family. All patients in the family were found to carry a heterozygotic missense variant in *CYCS* gene by whole exome sequencing (WES), while the pathogenicity of the genetic variation was also evaluated. According to the clinical and genetic features of the patients in the pedigree, thrombocytopenia 4 was diagnosed. This work could be a scientific evidence to support on thrombocytopenia diagnosis and its prognostic management.

## 2 Materials and Methods

### 2.1 Patient Clinical Information

**FIGURE 1:**
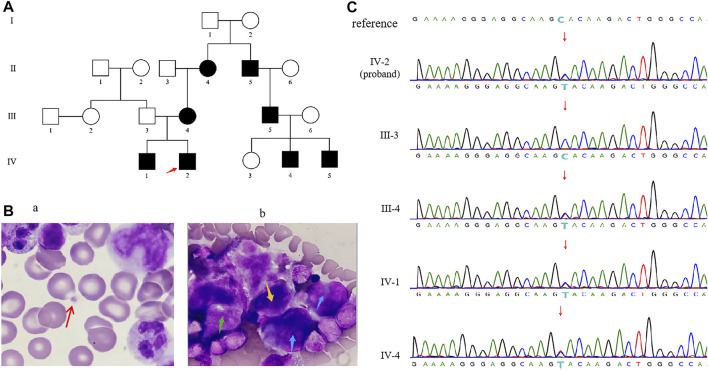
**(A)** Familial pedigree of CYCS-mutated thrombocytopenia. The red arrow indicates the proband. **(B)** Optical microscopy of bone marrow cells from the proband showed the number of platelets were reduced. The red arrow on the left **(Ba)** showed a single platelet was sporadically visible, and the blue arrow points to promegakaryocytes **(Bb)**, the green arrow points to granular megakaryocytes **(Bb)**, and the yellow arrow points to thromocytogenic megakaryocytes **(Bb)**. **(C)** Sanger sequencing of *CYCS* gene c.79C > T (p.His27Tyr) variant in genomic DNA from the family.

The probands (IV-2) was the second son of a non-consanguineous Chinese couple (Figure 1A). He was born at gestational age of 39 + 5 weeks by spontaneous vaginal delivery with normal birth history (birth weight, 3500 g). Multiple hemorrhagic spots were found on the facial and back skin of the proband at birth. After 7 days, he was admitted for further treatment. Physical examination was normal. Brain magnetic resonance imaging (MRI) showed mild cerebral hemorrhage in the left ventricle and focal white matter injury near the posterior horn of the left ventricle. The full blood count test yielded a low platelet count at 54 × 10^9^/L (reference range: 150–450 × 10^9^/L) and the increased leucocyte count at 30.68 × 10^9^/L (reference range: 10.4–12.21 × 10^9^/L). After antibiotics and immunoglobulin treatment, the hemorrhagic spots disappeared with normal leucocyte counts and improved platelet counts (ranges from 56 to 96 × 10^9^/L).

At 16 days of age, the patient showed no signs of hemorrhage with normal abdominal ultrasound and urine organic acids. Blood tandem mass spectrometry analysis showed camitine deficiency. Optical microscope of bone marrow showed megaloblasts and toxic granules below the stage of myelocyte and metamyelocyte, indicating as bone marrow infection. In addition, the whole bone marrow smear showed 550 megakaryocytes, among which promegakaryocytes accounted for 30%, granular megakaryocytes accounted for 62%, thromocytogenic megakaryocytes accounted for 8%. These abnormal proportions of megakaryocytes indicate that megakaryocytes maturation problem leaded to a decrease in platelet count (Figure 1B). Subsequently, he was instructed to continue antibiotics therapy and oral administration of levocarnitine. At 34 days of age, the patient showed no signs of hemorrhage. The results of the secondary MRI and blood tandem mass spectrometry analysis were normal. The platelet counts maintained at decreased levels in multiple regular examinations, one of which showed normal level at 108 × 10^9^/L (reference range: 150–450 × 10^9^/L).

Clinical features of his family members were summarized in supplemental Table 1, which details a history of thrombocytopenia in the proband’s maternal family member. The patient’s father is healthy. Of the 11 subjects, 8 presented low platelet counts (IV-2, IV-1, III-4, II-4, II-5, III-5, IV-4, IV-5), and the remaining subjects showed normal platelet level (III-3, III-6, IV-3). The platelet counts of IV-2, IV-1, III-4, and II-4 were examined multiple times, while the rest examined once. The studied family members, including the proband, had platelet counts ranging from 38 to 110 × 10^9^/L with a reference interval of 150–450 × 10^9^/L. The mean volumes and morphology of the sampled platelet were normal. Most studied patients had mild bleeding of skin mucosa, except IV-5 with epistaxis, and the proband showed the spontaneous skin bleeding (Supplemental Table 1). All the patients had no symptom of mitochondrial disease such as neurodegeneration, diabetes, myopathy, eye, and kidney diseases.

**TABLE 1:** Clinical features of the family members from the current study.

Case clinical features	IV-2 (proband)	IV-1	III-4	II-4	II-5	III-5	IV-4	IV-5	III-3	III-6	IV-3
Brusied more than normal	−	+	+	+	+	−	−	−	−	−	−
Epistaxis	−	−	−	−	−	−	−	+	−	−	−
Bleeding spot	+	−	−	−	−	−	−	−	−	−	−
Mitchondial disease	−	−	−	−	−	−	−	−	−	−	−
Platelet counts 150–450 × 10^9^/L)	54–108	89–110	41–45	50–70	60	38	98	76	200	205	217
MPV	N	N	N	N	N	N	N	N	N	N	N
Variant	*CYCS*: NM_018947.6:c.79C > T (p.His27Tyr), heterozygous								Wild-type		

N, normal; MPV, mean platelet volumes.

### 2.2 Sample Collection

This study was approved by the Ethics Committee of Xi’an Children’s Hospital, and written informed consent was obtained from each participant or their guardian(s). The peripheral blood (3 ml) was individually sampled from the proband and his family members and collected in EDTA anticoagulant tube.

### 2.3 Whole Exome Sequencing

1 μg genomic DNA was extracted from 200 µL peripheral blood using a Qiagen DNA Blood Midi/Mini kit (Qiagen GmbH, Hilden, Germany) following the manufacturer’s protocol. Library preparation was performed using NanoWES (Berry Genomics, China) according to the manufacturer’s Protocol. Novaseq6000 platform (Illumina, San Diego, United States) was used for sequencing. The exome sequencing was performed with a minimum median coverage of 80X. The sequencing reads were aligned to the human reference genome (hg38/GRCh38) using Burrows–Wheeler Aligner tool. Verita Trekker^®^ Variants Detection System by Berry Genomics and the third-party software GATK were employed for variant calling. Variants with lower quality (read depth < 10×, allele fraction < 30%) were eliminated. Variant annotation and interpretation were conducted by ANNOVAR (Wang et al., 2010) and the Enliven^®^ Variants Annotation Interpretation System authorized by Berry Genomics. All variants were filtered through population databases including the 1,000 Genomes Project (1000G), Exome Aggregation Consortium (ExAC), and gnomAD, only those variants with population frequencies less than 1/1,000 in all databases were counted. Variant pathogenicity/deleteriousness prediction was evaluated using SIFT, Poly-Phen_2, Mutation Taster, REVEL, FATHMM, CADD. Prediction of variant impact on splicing was evaluated by dbscSNV, Human Splicing Finder (HSF), and SpliceAI. To maximize clinically diagnostic yield, the known pathogenic variants from Human Gene Mutation Database and ClinVar (Landrum et al., 2016; Stenson et al., 2020) were also retained for further evaluation. The variants were classified to five categories “pathogenic,” “likely pathogenic,” “uncertain significance,” “likely benign,” and “benign”--according to the American College of Medical Genetics and Genomics (ACMG) guidelines for interpretation of genetic variants (Richards et al., 2015). The suspected SNV/Indels were validated using Sanger sequencing.

The three-dimensional (3D) structure of CYCS protein of the missense variant was analyzed by Swiss-PDBviewer (PDB:3ZOO).

## 3 Results

### 3.1 Genetic Analysis and Co-Segregation in the Family

**FIGURE 2:**
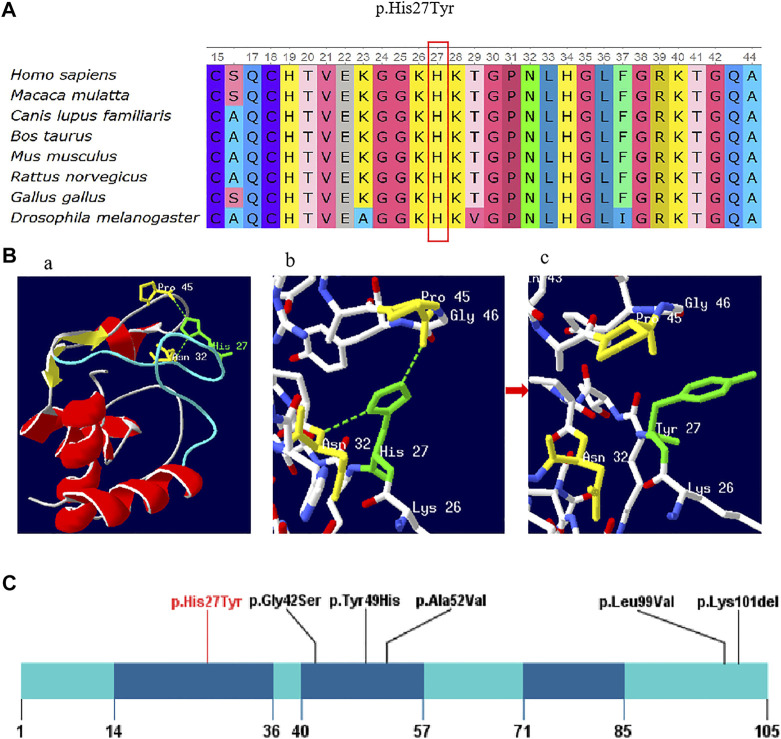
**(A)** Conservation of the p. His27Tyr variant across various species. **(B)** Amino acid and conformation changes of the p.His27Tyr polypeptide wild-type and mutant type. His27 is located in the random coil domain of CYCS by colored green **(Ba)**; Stick models shows the amino acids around His27 and the selected side chains, wild-type His27 forms a hydrogen bond (green dotted line) with Asn32 and Pro45 (colored yellow) respectively **(Bb)**, and the hydrogen bond was lost in mutant type Tyr27 **(Bc)**. **(C)** Schematic presentation of linear CYCS protein (NM_018947.6) with all variants, red font indicate reported variant in the present study. The dark blue indicates three Ω-loops.

A heterozygous variant in the *CYCS* (NM_018947.6: c.79C > T (p.His27Tyr) was identified for the proband by WES. Sanger sequencing further confirmed that all affected members (IV-2, IV-1, III-4, II-4, II-5, III-5, IV-4, IV-5) carried the same heterozygous variant, while other studied members (III-3, III-6, IV-3) did not carry it (Figure 1C). So, the variant segregated with the disorder in this family (PP1_Strong). This novel variant has not been reported in previous literature, and the c.79C > T substitution was not seen in gnomAD database (PM2_Supporting). Sequence alignment of CYCS protein among multiple species showed that p.His27 is highly conserved across evolution (Figure 2A), suggesting that His27 could play a vital role in maintaining the stability and function of proteins. The results of multiple statistical methods (REVEL) predicted that the variant could cause detrimental effect on gene function (PP3). According to the ACMG guidelines, c.79C > T (p.His27Tyr) is defined as “Likely Pathogenic”. This novel variant has been submitted to ClinVar with the variation ID: 1210164.

### 3.2 Structure-Function Correlations of *CYCS* Variants

The impact of this missense variant was also evaluated by examining the 3D structural viewpoint of the variant protein. Our study mapped the mutation position onto the crystal structure of human cytochrome c (PDB:3ZOO). As shown in Figure 2B, p.His27Tyr variant is located in the random coil domain of CYCS (Figure 2Ba), which may affect the global conformation and activity of protein. When His27 is replaced by Tyr, the hydrogen bonds of His27 interacting with Asn32 and Pro45 were broken, and the amino acid side chain is changed from imidazole to benzene (Figures 2Bb,Bc).

## 4 Discussion

In this study, we firstly reported a 3-generation family with autosomal dominant non-syndromic thrombocytopenia in China. The whole exome sequencing identified a novel missense heterozygous variant c.79C > T (p.His27Tyr) of *CYCS* gene in the pedigree with eight patients (IV-2, IV-1,III-4, II-4, II-5, III-5, IV-4, IV-5). The clinical features of the studied patients were consistent with that from other reports (Morison et al., 2008; De Rocco et al., 2014; Johnson et al., 2016). More, they had normal fertility and longevity, with no evidence of mitochondriopathies (Finsterer, 2004), while other family members (III-3, III-6, IV-3) of the study did not carry the variant. It was assumed that the variation p. His27Tyr of *CYCS* could be the genetic etiology of autosomal dominant non-syndromic thrombocytopenia in this family case. The finding enriches the mutation spectrum of *CYCS* gene and laid a foundation for future studies on the correlation between genotype and phenotype. This conclusion may help patients to prevent the disease risk associated with thrombocytopenia and help clinicians to do differential diagnoses including acquired and inherited forms.

In 2008, Morison *et al.* reported the first variant of the *CYCS* gene identified in a family with thrombocytopenia (Morison et al., 2008). Globally, total five variants have been reported to be associated with thrombocytopenia, including four missense variant (p.Gly42Ser, p.Tyr49His, p.Ala52Val, and p.Leu99Val) and one small deletion variant p.Lys301del (in-frame) (De Rocco et al., 2014; Johnson et al., 2016; Uchiyama et al., 2018; Turro et al., 2020). The p. His27Tyr variant found in this study was the fifth missense mutation. To date, multiple studies has investigated the missense mutation effect on physical structure and bio-function of CYCS protein. Liptak et al. demonstrated that the proapoptotic Gly42Ser mutation altered the heme electronic structure and increased the rate of electron self-exchange, resulting in the enhanced proapoptotic activity of Gly42Ser (Liptak et al., 2011). De Rocco *et al.* reported that the p. Gly42Ser and p.Tyr49His variants in yeast and mouse cellular models were responsible of the diminished respiratory level and increased apoptotic rate (De Rocco et al., 2014). Lei et al. showed that the Ala51Val variant enhanced peroxidase activity by destabilizing the native state of Cyt-c, and all three variants Gly42Ser, Tyr49His, and Ala51Val had reduced global and local stability than that of wild type Cyt-c (Lei and Bowler, 2019). Moreover, Uchiyama *et al.* provided that the mutation of p.Lys301del could significantly reduced cytochrome c protein expression and cause functional defects in the mitochondrial respiratory chain (Uchiyama et al., 2018). The existing findings are not enough to explain the molecular mechanism of thrombocytopenia, however, they could be used as scientific evidence for thrombocytopenia diagnosis.

Cyt-c, a highly evolved protein in different species (St-evens, 2011), contains three highly conserved Ω-loops including residues 14–36, 40–57, and 71–85 (Karsisiotis et al., 2016). Ω-loops play an important role in maintaining the protein function and stability (Fetrow, 1995). Of the five reported missense variants, three variants p.Gly42Ser, p.Tyr49His, and p.Ala52Val were located in the second Ω-loop domain (residues 40–57), two variants p.Leu99Val, and p.Lys301del were located in the C-terminal of the protein. The p.His27Tyr variant found in this study was in the first Ω-loop (residues 14–36) (Figure 2C), which may affect the stability of Cyt-c. Previous studies have reported that His27 in the wild type Cyt-c interacted with Pro45 through an hydrogen bond which is essential for sustaining the orientation of the heme conformation and the α-helices, inflicting the cardiolipin binding to cyt-c and subsequent apoptotic events (Balakrishnan et al., 2012). Additionally, the 3D structure of CYCS protein was predicted and it showed that the amino acid substitution (His27Tyr) could lead to the cleavage of hydrogen bonds between His27 and Pro45, causing the instability of the protein structure and destruction of its biological function. For mutant protein, the positive charged Histidine was replaced by the neutral Tyrosine, which negatively influence the electron transport in mitochondrial oxidative respiratory chain. Hence, we assumed that the variant p.His27Tyr could be the genetic etiology of the thrombocytopenia in the family case. Nevertheless, the genetic effect of this variant needs to be verified in future study.

In summary, with the rapid development of molecular biotechnology, genetic analysis has been widely used in rare disease diagnosis. Our findings demonstrated that a new missense variant of the *CYCS* gene was associated with non-syndromic thrombocytopenia identified by WES. Further research is required to understand the impact of *CYCS* variants on changing platelet production. The outcome could be beneficial for thrombocytopenia diagnosis and prognostic management.

## Data Availability

The datasets presented in this article are not readily available due to ethical and privacy restrictions. Requests to access the datasets should be directed to the corresponding author.
